# *Vibrio aphrogenes* sp. nov., in the Rumoiensis clade isolated from a seaweed

**DOI:** 10.1371/journal.pone.0180053

**Published:** 2017-06-29

**Authors:** Mami Tanaka, Shoko Endo, Fumihito Kotake, Nurhidayu Al-saari, A. K. M. Rohul Amin, Gao Feng, Sayaka Mino, Hidetaka Doi, Yoshitoshi Ogura, Tetsuya Hayashi, Wataru Suda, Masahira Hattori, Isao Yumoto, Toko Sawabe, Tomoo Sawabe, Toshiyoshi Araki

**Affiliations:** 1 Laboratory of Microbiology, Faculty of Fisheries, Hokkaido University, Hakodate, Japan; 2 Process Development Laboratories, Research Institute for Bioscience Products & Fine Chemicals, Ajinomoto Co.Inc., Kawasaki, Japan; 3 Department of Bacteriology, Faculty of Medical Sciences, Kyushu University, Fukuoka, Japan; 4 Laboratory of Metagenomics, Graduate School of Frontier Sciences, University of Tokyo, Kashiwa, Japan; 5 Department of Microbiology and Immunology, Keio University School of Medicine, Tokyo, Japan; 6 Graduate School of Advanced Science and Engineering, Waseda University, Tokyo, Japan; 7 Bioproduction Research Institute, National Institute of Advanced Industrial Science and Technology, Sapporo, Japan; 8 Department of Food and Nutrition, Hakodate Junior College, Hakodate, Japan; 9 Iga Research Institute of Mie University, Iga, Japan; UFRJ, BRAZIL

## Abstract

A novel strain *Vibrio aphrogenes* sp. nov. strain CA-1004^T^ isolated from the surface of seaweed collected on the coast of Mie Prefecture in 1994 [1] was characterized using polyphasic taxonomy including multilocus sequence analysis (MLSA) and a genome based comparison. Both phylogenetic analyses on the basis of 16S rRNA gene sequences and MLSA based on eight protein-coding genes (*gapA*, *gyrB*, *ftsZ*, *mreB*, *pyrH*, *recA*, *rpoA*, and *topA*) showed the strain could be placed in the Rumoiensis clade in the genus *Vibrio*. Sequence similarities of the 16S rRNA gene and the multilocus genes against the Rumoiensis clade members, *V*. *rumoiensis*, *V*. *algivorus*, *V*. *casei*, and *V*. *litoralis*, were low enough to propose *V*. *aphrogenes* sp. nov. strain CA-1004^T^ as a separate species. The experimental DNA-DNA hybridization data also revealed that the strain CA-1004^T^ was separate from four known Rumoiensis clade species. The G+C content of the *V*. *aphrogenes* strain was determined as 42.1% based on the genome sequence. Major traits of the strain were non-motile, halophilic, fermentative, alginolytic, and gas production. A total of 27 traits (motility, growth temperature range, amylase, alginase and lipase productions, and assimilation of 19 carbon compounds) distinguished the strain from the other species in the Rumoiensis clade. The name *V*. *aphrogenes* sp. nov. is proposed for this species in the Rumoiensis clade, with CA-1004^T^ as the type strain (JCM 31643^T^ = DSM 103759^T^).

## Introduction

The genus *Vibrio*, first proposed in 1854, is a large group of bacteria showing Gram negative and with most species requiring salt for growth [2]. Currently 111 *Vibrio* species have been described (http://www.bacterio.net/) [2]. The genus *Vibrio*, along with other members of *Vibrionaceae*, is at the forefront of bacterial taxonomy, having been tested using new methodologies, e.g. amplified fragment length polymorphism (AFLP), multilocus sequence analysis (MLSA), and genome-based sequence comparison [2–6]. Among them, the MLSA has been used as a powerful tool to find “clades” sharing a possible common ancestry among metabolically versatile *Vibrionaceae* species/strains [3–5]. The 8-gene MLSA defines 23 *Vibrio* and *Photobacterium* clades and an *Enterovibiro*-*Grimontia*-*Salinivibrio* super clade, which help us to elucidate the dynamic nature of biodiversity and evolutionary history interacting with the Earth’s ecosystem [5]. Rapid expansion of genome sequencing methodology in bacterial taxonomy also assists and accelerates the accumulation of our knowledge of vibrio biodiversity and has contributed towards the proposals for new clades within the family *Vibrionaceae* such as Agarivorans [3] and Swingsii [7].

*Vibrio rumoiensis* was isolated as a strong catalase producer from the drain pool of a fish processing plant that uses H_2_O_2_ as a bleaching and microbial agent [8]. In one of the first uses of MLSA for *Vibrionaceae* taxonomy, *V*. *rumoiensis* was classified as an orphan clade species [4]. Subsequent MLSA showed that *V*. *litoralis*, a tidal flats isolate [9], could share a common ancestry with *V*. *rumoiensis* which led to the proposal for the Rumoiensis clade [5]. Currently, there are four species known to be a member of the Rumoiensis clade: *V*. *rumoiensis*, *V*. *algivorus* [10], *V*. *casei* [11], and *V*. *litoralis* [9]. *V*. *casei*, and *V*. *algivorus* were isolated from surface of cheeses and the gut of a turban shell, *Turbo cornutus*, respectively. All species share an assimilation pattern of carbohydrates such as D-mannose, D-galactose, D-fructose, and D-mannitol, nitrate reduction, and, with the exception of *V*. *casei*, non-motility [9–11]. The ecophysiological coherence of Rumoiensis clade species is still unknown.

A vibrio strain phylogenetically related to the Rumoiensis clade was isolated from the surface of seaweed samples collected from the coast of Mie prefecture, Japan in 1994. This bacterium was originally isolated as a κ-carrageenase producer with a *cgk* gene [1]. Further phylogenetic, genetic and genomic characterizations in this study revealed the novelty of the strain placing it into the Rumoiensis clade. Importantly, the strain is the first microbe to produce hydrogen from alginate. The gas production is supported by having a *hyf*-type formate hydrogen lyase gene cluster, the discovery of which is the first in the gas producing species in the Rumoiensis clade. The *V*. *aphrogenes* sp. nov. CA-1004^T^ might hold important clues in elucidating the evolutionary history of species in the Rumoiensis clade and a biotechnological novelty in *Vibrionaceae*.

## Materials and methods

### Bacterial strains and phenotypic characterization

*V*. *aphrogenes* strain CA-1004^T^ isolated from seaweed surface in 1994 collected at Mie Prefecture in Japan [1] was characterized. For phenotypic characterization, all type strains belonging to the Rumoiensis clade were cultured on ZoBell 2216E agar medium and the phenotypic characteristics were determined according to previously described methods [3].

### Phylogenetic analysis based on a 16S rRNA gene

**Fig 1:**
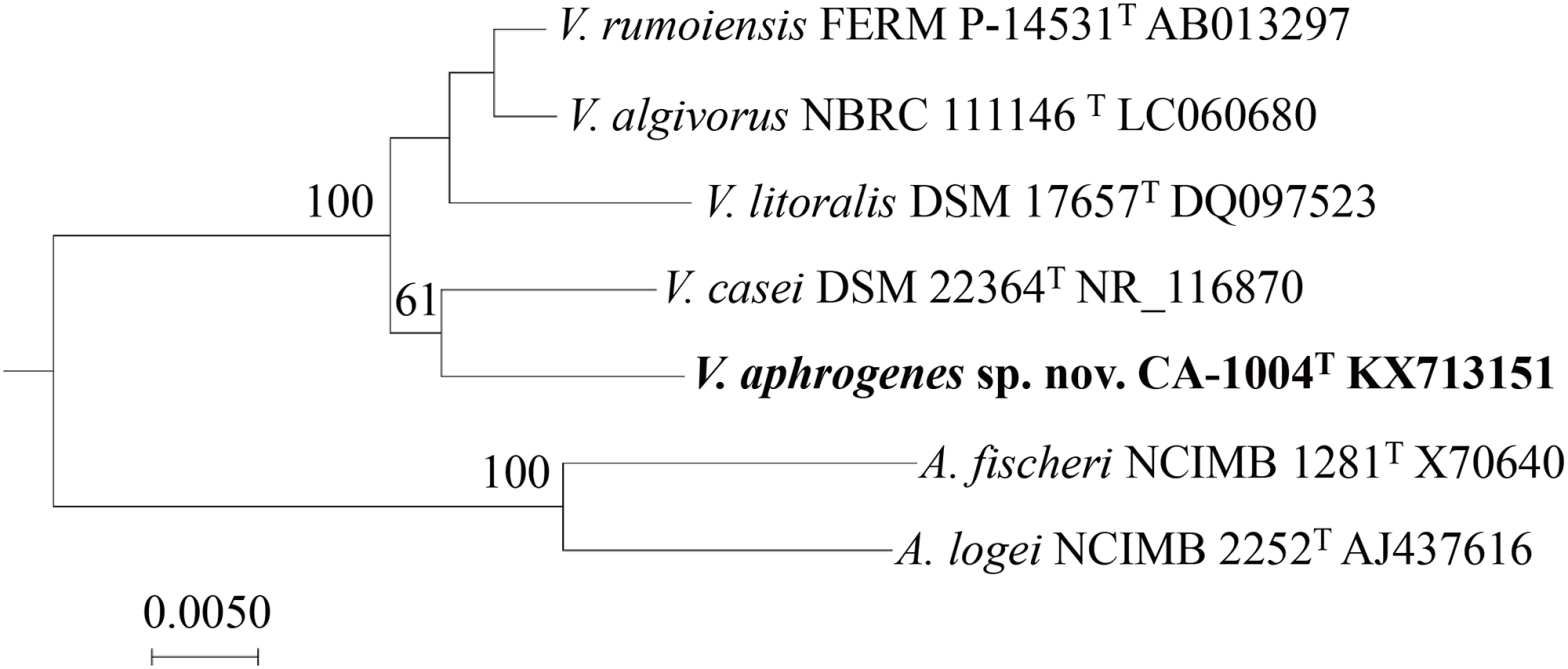
A rooted phylogenetic tree on the basis of 16S rRNA gene sequences. This figure combines the results of three analyses, i.e. neighbor-joining (NJ), maximum-parsimony, and maximum-likelihood. The topology shown was obtained by NJ with 500 bootstrap replications. The bootstrap value was only indicated on branches supported by three methods.

A 1400 bp of 16S rRNA gene sequence of the strain CA-1004^T^ was obtained according to Al-saari et al. [3], using the amplification primers (24F and 1509R) corresponding to positions 25 to 1521 in the *Escherichia coli* sequence. The other *Vibrionaceae* sequences used to reconstruct a broad phylogenetic tree shown in S1 Fig were retrieved from the GenBank/DDBJ/EMBL database and analyzed using ClustalX version 2.1 [12] and MEGA version 7.0.16 programs [13]. In the final tree (Fig 1), the 16S rRNA gene sequences of *A*. *fischeri* NCIMB 1281^T^ (X70640), *A*. *logei* NCIMB 2252^T^ (AJ437616), *V*. *algivorus* NBRC 111146^T^ (SA2^T^) (LC060680), *V*. *casei* DSM 22364^T^ (NR_116870), *V*. *litoralis* DSM 17657^T^ (DQ097523), and *V*. *rumoiensis* FERM P-14531^T^ (S-1^T^) (AB013297) were used to reconstruct the tree using the MEGA program with three different methods; neighbor-joining (NJ), maximum parsimony (MP), and maximum likelihood (ML). The robustness of each topology was checked using the NJ method with 500 bootstrap replications. Evolutionary distances of the NJ method were corrected using the Jukes-Cantor method.

### Genome sequencing

Draft genome sequences of strain CA-1004^T^, *V*. *algivorus* NBRC 111146^T^, *V*. *casei* DSM 22364^T^, and *V*. *rumoiensis* FERM P-14531^T^ were obtained using the MiSeq platform. For CA-1004^T^ only, a paired-end library and an 8 kb mate-pair library were constructed using the Nextera XT DNA Library Preparation Kit and the Nextera Mate Pair Sample Preparation Kit, respectively. Genome sequences of the other strains were obtained from a paired-end library preparing using the Nextera XT DNA Library Preparation Kit for *V*. *aphrogenes* and TruSeq PCR-Free kit for *V*. *algivorus*, *V*. *casei* and *V*. *rumoiensis*. The genome sequence was assembled using Platanus [14]. The sequence was deposited in the DDBJ/GenBank/EMBL database under accession numbers described below.

### Multilocus sequence analysis (MLSA)

**Fig 2:**
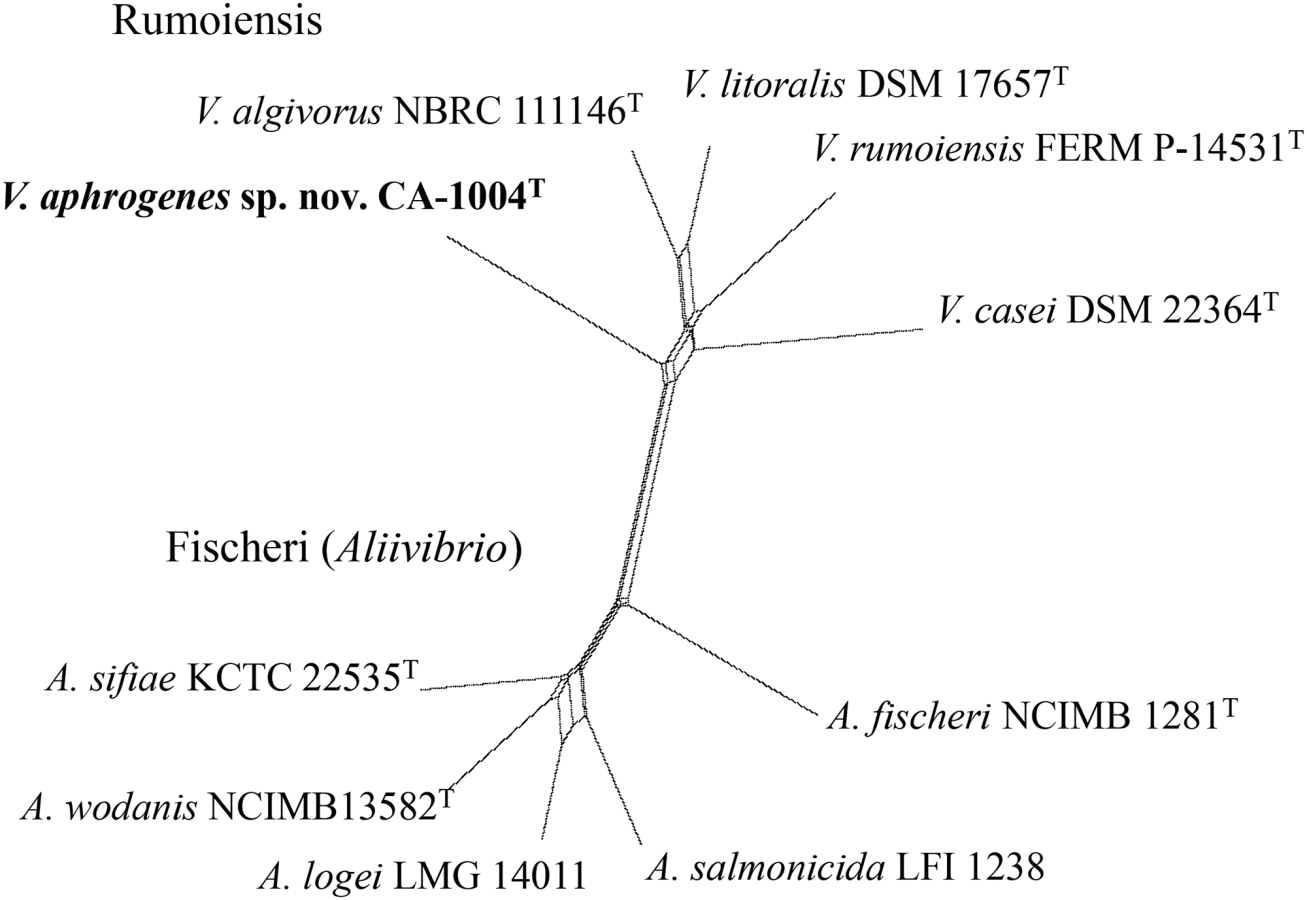
Concatenated split network tree based on eight gene loci. The *gapA*, *gyrB*, *ftsZ*, *mreB*, *pyrH*, *recA*, *rpoA*, and *topA* gene sequences were concatenated including the representative of the *Vibrio aphrogenes* sp. nov. strain CA-1004^T^. Phylogenetic tree was generated using the SplitsTree4 program.

Sequences of eight protein-coding genes (*ftsZ*, *gapA*, *gyrB*, *mreB*, *pyrH*, *recA*, *rpoA*, and *topA*) of CA-1004^T^ were retrieved from the genome sequences. MLSA was conducted in the same manner as previously described [4–5]. The sequences were aligned using ClustalX 2.1 [12]. The domains used to construct the tree shown in Fig 2 were regions of the *ftsZ*, *gapA*, *gyrB*, *mreB*, *pyrH*, *recA*, *rpoA*, and *topA* genes; positions 196–630, 226–861, 442–1026, 391–895, 175–543, 430–915, 385–762, and 571–990 (*V*. *cholerae* O1 Eltor N16961 (AE003852) numbering), respectively. The MEGA program was used to calculate the sequence similarity. Split decomposition analysis (SDA) was performed using SplitsTree version 4.14.3 with a neighbor net drawing and a Jukes-Cantor correction [15–16]. Each aligned set was concatenated and used to reconstruct the network.

### DNA-DNA hybridizations

Strains used for DNA-DNA hybridization were CA-1004^T^, *V*. *algivorus* NBRC 111146^T^, *V*. *casei* DSM 22364^T^, *V*. *litoralis* DSM 17657^T^, and *V*. *rumoiensis* FERM P-14531^T^. DNAs of the strains were prepared accordingly to Marmur [17] with minor modifications. DNA-DNA hybridization experiments were performed using the fluorometric direct binding method in microdilution wells described previously [3]. In brief, the DNAs of CA-1004^T^ were labeled with photobiotin (Vector Laboratories, Inc., Burlingame, CA). After immobilization of unlabeled single stranded DNA of CA-1004^T^ in microdilution wells (Immuron 200, FIA/LIA plate, black type, Greiner labotechnik, Germany), hybridization was performed under optimal conditions using the CA-1004^T^ labeled DNA as a probe following pre-hybridization [3]. Detection of the hybridized probe was performed using fluorometry (Infinite 200, Tecan, Switzerland) after binding streptavidin-β-galactosidase to the probe DNA. 4-Methylumbelliferyl-β-d-galactopyranoside (6 x 10^−4^ M; Wako, Osaka, Japan) was used for a fluorogenic substrate for β-galactosidase. DNA-DNA homology was calculated according to the previous report [3] based on an average value measured from three wells.

### Genome analysis and in silico DNA-DNA similarity calculation

General genome features including DNA G+C content were determined using the Rapid Annotations Using Subsystems Technology (The RAST server version 4.0) [18]. *In silico* DDH values from Genome-to-Genome Distance Calculator (GGDC 2) [19–20] and Average Nucleotide Identity (ANI) values of CA-1004^T^ against *V*. *algivorus* NBRC 111146^T^, *V*. *casei* DSM 22364^T^, *V*. *litoralis* DSM 17657^T^, and *V*. *rumoiensis* FERM P-14531^T^ were estimated using Orthologous Average Nucleotide Identity Tool version 0.93 [21]. Comparison of genes encoding the *hyf*-type formate hydrogen lyase complex and the flanking region was performed using GenomeTraveler (In Silico Biology, Inc., Yokohama, Japan).

### Hydrogen production from alginate

Strain CA-1004 was cultured at 25°C in a 100 mL marine broth (0.5% (w/v) polypeptone, 0.1% (w/v) yeast extract) containing 100 mM MES (Dojindo, Kumamoto, Japan), supplemented with 1.0% (w/v) sodium alginate. A 3.0% (w/v) mannitol supplemented marine broth was used as a positive control. The pH of the medium was maintained at 6.0 using a pH controller (DT-1023P, ABLE, Tokyo, Japan) equipped with an autoclavable electrode (FermProbe pH electrodes, Broadley-James Corp., Branford, USA) by adding 5 N NaOH or HCl. Biogas was captured in an aluminium bag, and the H_2_ gas production was determined using gas chromatography (GC2014 Shimadzu, Kyoto, Japan) with a thermal conductivity detector and a ShinCarbon ST column (Shinwa Chemical Industries Ltd., Kyoto, Japan).

### Nucleotide sequence accession number

The genome sequence data for CA-1004^T^, *V*. *algivorus* NBRC 111146^T^, *V*. *casei* DSM 22364^T^, and *V*. *rumoiensis* FERM P-14531^T^ were deposited at DDBJ/EMBL/GenBank under the accession number BDGR01000001-BDGR01000024, BDSC01000001-BDSC01000008, BDSD01000001-BDSD01000055, and BDSE01000001-BDSE01000047, respectively. The 16S rRNA gene sequence of CA-1004^T^ was deposited in GenBank under KX713151.

## Results and discussion

The phylogenic analysis based on 16S rRNA gene sequences showed the strain CA-1004^T^ is a member of the genus *Vibrio* (S1 Fig): more precisely, the strain was closely related to members of the Rumoiensis clade with a high bootstrap support [4–5] (Fig 1). Sequence similarities of the 16S rRNA gene against those of Rumoiensis clade species, *V*. *algivorus* NBRC 111146^T^, *V*. *rumoiensis* FERM P-14531^T^, *V*. *casei* DSM 22364^T^, and *V*. *litoralis* DSM 17657^T^ were 98.4%, 98.0%, 97.9%, and 96.8%, respectively. These levels of similarity are below or in the proposed threshold range for the species boundary, 98.2–99.0% [22,23,24]. To further confirm the genetic coherence, DNA-DNA similarity of CA-1004^T^ against Rumoiensis clade species was experimentally measured. Using CA-1004^T^ as a labelled strain, DDH values against *V*. *algivorus* NBRC 111146^T^, *V*. *casei* DSM 22364^T^, *V*. *litoralis* DSM 17657^T^, and *V*. *rumoiensis* FERM P-14531^T^ were 15.4%, 12.0%, 4.9%, and 5.6%, respectively. These DDH values were sufficiently below the species boundary (<70%) to propose CA-1004^T^ as a new species in the Rumoiensis clade. MLSA using eight protein-coding genes also showed the clear separation of the Rumoiensis clade containing the CA-1004^T^ from the other clades of *Vibrionaceae* species, which suggests a common ancestry of the CA-1004^T^ and the other Rumioensis clade species (Fig 2, S2 Fig).

**Fig 3:**
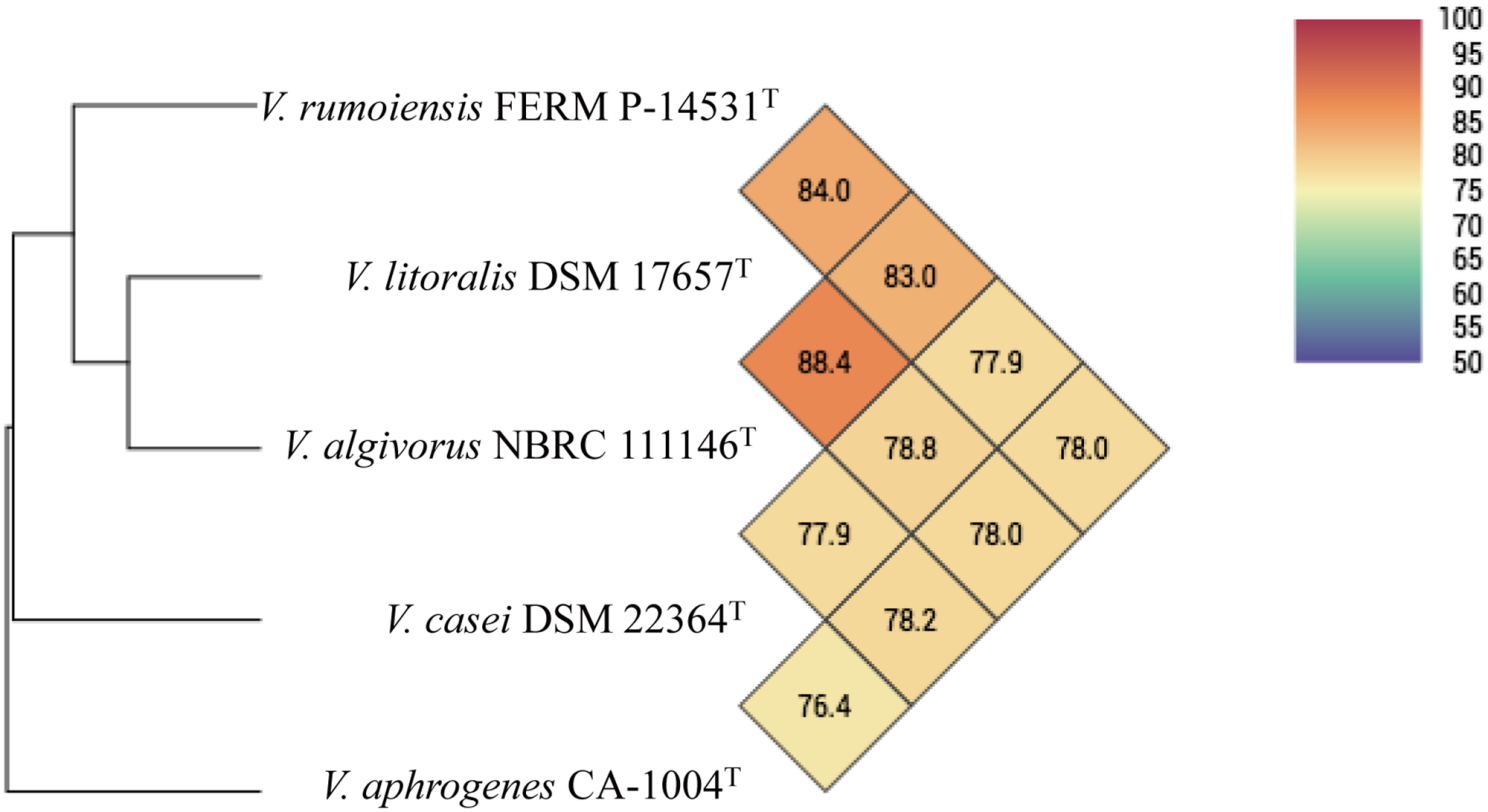
Heatmap generated with OrthoANI values calculated from Orthologous Average Nucleotide Identity Tool version 0.93 [21].

*In silico* genome comparison with CA-1004^T^ was also performed using the draft genomes of *V*. *algivorus* NBRC 111146^T^, *V*. *casei* DSM 22364^T^, *V*. *litoralis* DSM 17657^T^, and *V*. *rumoiensis* FERM P-14531^T^. The *in silico* DDH values (Formula 2, recommended) of CA-1004^T^ against *V*. *algivorus* NBRC 111146^T^, *V*. *casei* DSM 22364^T^, *V*. *litoralis* DSM 17657^T^, and *V*. *rumoiensis* FERM P-14531^T^ were 19.5%, 20.9%, 20.2%, and 20.5%, respectively, further distinguishing CA-1004^T^ from these species. Average nucleotide identity (ANI) was also calculated using the draft genome sequences, and the ANI values of CA-1004^T^ against *V*. *algivorus* NBRC 111146^T^, *V*. *casei* DSM 22364^T^, *V*. *litoralis* DSM 17657^T^ and *V*. *rumoiensis* FERM P-14531^T^ were 78.2%, 76.4%, 78.0%, and 78.0%, respectively (Fig 3), much below the species threshold of 95–96% for species circumscription [6,21,25–26]. These values of experimental DDH, *in silico* DDH, and ANI, along with phylogenic analyses, showed that CA-1004^T^ should be considered as a new species, indicating less genetic cohesion of CA-1004^T^ to the other Rumoiensis clade species.

On the basis of concatenated sequences of eight MLSA protein-coding genes, CA-1004^T^ is likely to share a common ancestry with members of the Rumoiensis clade. This was confirmed by comparative analysis of phenotypic and biochemical features of CA100-4^T^ with other members of the Rumoiensis clade (Table 1), showing some degree of phenotypic coherence between the different species. They grow at temperatures between 4 and 30°C, require salt for growth, and tested positive for nitrate reduction, oxidase, catalase, DNase, and alginase production. They were negative for growth on TCBS agar, lysine and ornithine decarboxylation, acetoin production, and indole production. On the other hand, CA-1004^T^ was distinguished from the close neighbors by several traits, such as showing positive results for gas production from D-glucose and arginine dihydrolase. Apparent κ-carrageenase activity reported by Araki et al. [1] was detected in the type strain proposed, but no any κ-carrageenase activities or *cgk* genes were retained in the draft genome sequence. The genes may have been lost during the long term serial transfers. The five species belonged into the Rumoiensis clade can grow wide range of NaCl concentration (Table 1).

**Table 1:** Phenotypic characteristics for distinguishing *Vibrio aphrogenes* sp. nov. and their closely related species. Taxa are indicated as: (1) *V*. *aphrogenes* CA-1004^T^, (2) *V*. *algivorus* NBRC 111146^T^, (3) *V*. *casei* DSM 22364^T^, (4) *V*. *liotralis* DSM 17657^T^, (5) *V*. *rumoiensis* DSM 19141^T^.

Characteristics	1	2	3	4	5
Motility	−	−	+	−	−
Growth at					
37°C	+	+	−	+	+
40°C	+	+	−	−	w
Production of					
Amylase	−	−	+	−	+
Alginase	+	+	−	−	−
Lipase	+	−	+	−	+
Arginine dihydrolase	+	−	−	−	−
Gas production from D-glucose	+	−	−	−	−
Utilization of					
D-Fructose	−	+	+	+	+
Sucrose	−	−	+	+	−
Maltose	+	−	+	+	−
Melibiose	−	−	+	−	+
Lactose	−	−	+	−	+
D-Gluconate	+	+	+	−	+
Xylose	d	−	+	+	+
Glucronate	−	−	−	−	+
D-Glucosamine	+	−	+	−	+
Cellobiose	−	−	+	−	−
Propinonate	−	+	+	+	+
Arabinose	−	−	+	+	+
Glycerate	−	+	+	+	+
D-Raffinose	−	−	+	−	+
Rhamnose	−	−	+	+	−
D-Ribose	+	+	+	+	−
Salicine	−	−	+	−	+
L-Arginine	−	−	+	−	−
Histidine	−	−	+	−	−
L-Ornithine	−	−	+	−	−

All species were Gram negative, fermentative, require salt for growth, and oxidase- and catalase-positive. All species were positive for growth on 4, 15, 25, 30°C, growth in 1, 3, 6, 8, 10% NaCl broth, DNase, nitrate reduction, and utilization of D-mannose, D-galactose, fumarate, D-mannitol, glycerol, acetate, pyruvate, L-proline, L-alanine, L-asparagine, and L-serine. All species were negative for pigmentation, growth on TCBS, acetoin production, indole production, agarase, gelatinese, κ-carrageenase, lysine and ornithine decarboxylase, bioluminescence, and utilization of N-acetylglucosamine, succinate, citrate, aconitate, γ-aminobutyrate, L-tyrosine, D-sorbitol, DL-malate, amygdalin, α-ketoglutarate, trehalose, δ-aminovarate, L-glutamate, putrescine, D-galacturonate, DL-lactate, L-citrulline, and glycine.

**Fig 4:**
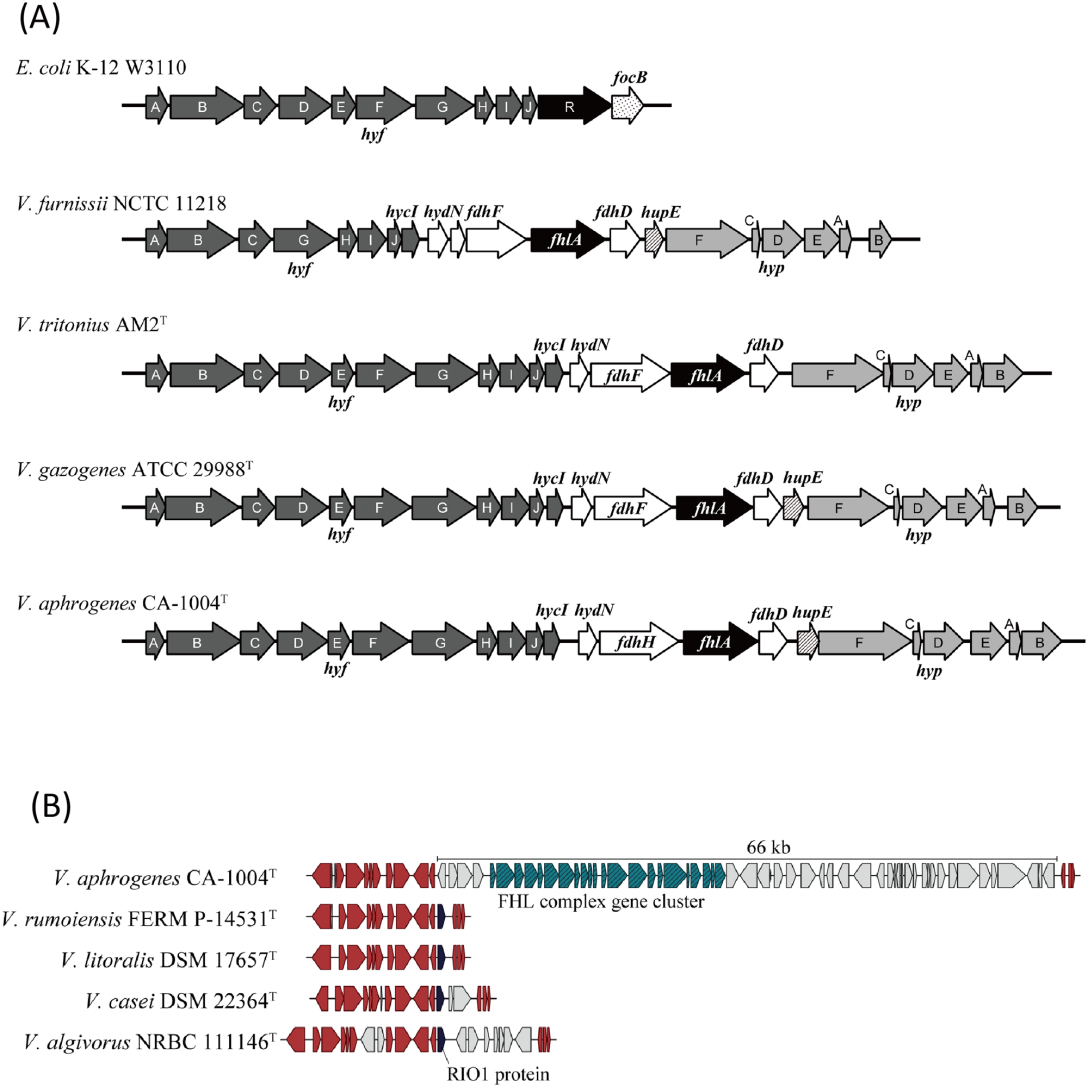
Comparison of *hyf* type formate hydrogen lyase (FHL) complex gene cluster. (A) Comparison of the FHL gene cluster of *Vibrio aphrogenes* sp. nov strain CA-1004 and those from *Escherichia coli* K-12, *Vibrio furnissii* NCTC 11218, *Vibrio tritonius* AM2^T^, and *Vibrio gazogenes* ATCC 29988^T^ (B) Comparison of the FHL gene cluster and the flanking region of *Vibrio aphrogenes* CA-1004^T^ to those of *V*. *algivorus* NBRC 111146^T^, *V*. *casei* DSM 22364^T^, *Vibrio litoralis* DSM 17657^T^, and *V*. *rumoiensis* FERM P-14531^T^. FHL complex gene cluster and RIO1 genes are shown in green and black, respectively. Genes shared among all genomes are represented in red. Genes shown in gray are unique genes in each strain.

Interestingly, the strain CA-1004^T^ possessed an entire gene set responsible for a *hyf*-type formate hydrogen lyase complex [27] (Fig 4A). The gene cluster consisted of genes for a major part of FHL (a hydrogenase complex and a formate dehydrogenase), and the *flhA* activator gene, which corresponds to the FHL-Hyp gene cluster of *V*. *tritonius* AM2^T^ [27]. The presence of the gene cluster supports the gas production phenotype of the strain. In addition to the gene cluster, a possible nickel transporter gene, *hupE*, was located between the formate dehydrogenase gene and the *hyp* gene cluster (Fig 4A). The *V*. *aphogenes*-type of FHL gene cluster involving the *hupE* gene is also found in Gazogenes clade species including *V*. *gazogenes* (Fig 4A) but the hydrogen productions by these strains are rather low (unpublished data). The biochemistry and molecular biology of HupE in the *V*. *aphrogenes* CA-1004^T^ have not been investigated yet, but the function of the *R*. *leguminosarum* HupE is recently identified as an energy-independent and specific diffusion facilitator for nickel transport for hydrogenase synthesis, on the basis of the kinetics using inhibitors and uncouplers such azide, arsenate, CCCP, and DCCD in the *Rhizobium* hydrogen uptake system [28–29]. Mutant assays also revealed good correlation between the nickel transport and the hydrogenase activity in *R*. *leguminosarum* [29]. The hydrogenase of *V*. *aphrogenes* is predicted as a [NiFe] hydrogenase on the basis of the primary structure comparison possessing CxxC motif required for [NiFe] center construction [27], the nickel transport via the HupE could have a strong link to the hydrogenase activity. Further genome comparison with other members of Rumoiensis clade revealed no any FHL-*fdhF*-*hyp* gene cluster. Other members of Rumoiensis possessed regions containing serine/threonine protein kinase, RIO1 family protein gene, replacing 66-kb genome region with FHL-*fdhF*-*hyp* gene cluster in *V*. *aphrogenes* (Fig 4B). As the gas production is an atypical phenotype not only in the genus *Vibrio* but also in the family *Vibrionaceae* [2,4–5,30], this might give important clues in revealing the evolutionary history of *hyf*-type gene cluster present in vibrios.

More interestingly, we found a direct hydrogen production (2.9 mL H_2_ gas at 25°C at 48 hours) by the *V*. *aphrogenes* strain CA-1004^T^ from alginate, which is major polysaccharide in brown seaweed. As alginate is known as one of the most oxidized polysaccharides, reduced fermentation products such as ethanol and lactate are unlikely to be produced from such substrate during the fermentation of bacteria due to the redox imbalance [31–32]. Since H_2_ is also known to be a reduced fermentation gaseous product, no bacteria possessing direct alginate-H_2_ conversion metabolisms have been reported until now. The new findings of the *V*. *aphrogenes* sp. nov. could illuminate the future metabolic pathway designs in H_2_ production even when using redox imbalanced substrates. We need further characterization to show how direct H_2_ production from alginate is controlled genetically and/or biochemically in this unique *Vibrio* species for future applications of *V*. *aphorogenes*.

In conclusion, polyphasic taxonomy with a genome-based strategy indicated *V*. *aphrogenes* as a new species in the genus *Vibrio*. Both 16S rRNA gene sequences phylogeny and MLSA based on eight protein-coding gene sequences placed the stain CA-1004^T^ into the Rumoiensis clade. Comparison of phenotypic features also places *V*. *aphrogenes* CA-1004^T^ in the genus *Vibrio*, while supporting its novelty (Table 1). The name *V*. *aphrogenes* is proposed to show its gas-producing features. Unfortunately we have only one strain of *V*. *aphrogenes* today, further ecological study is necessary for understanding the biodiversity and ecophysiological roles of the *V*. *aphrogenes* strains.

### Description of *Vibrio aphrogenes* sp. nov.

*V*. *aphrogenes* sp. nov. (aph.ro'ge.nes. Gr. n. aphros, foam; Gr. suff. -genes, producing; N.L. adj. aphrogenes, foam-producing, referring to gas formation of the strain)

Gram-negative, facultative anaerobic, non-motile rods isolated from surface of seaweed collected in Mie Prefecture in Japan. Colonies on ZoBell 2216E agar medium were cream or transparent white, round, and smooth on the edge. No flagellum was observed. Sodium ion is essential for growth. Growth occurs at NaCl concentrations of 1.0 to 10.0% and at temperatures between 4 and 40°C. *V*. *aphrogenes* tested positive for production of alginase, lipase and DNase, oxidase, catalase, gas production from D-glucose, arginine dihydrolase, and is able to assimilate D-glucose, D-mannitol, D-mannose, D-galactose, maltose, D-gluconate, fumarate, glycerol, acetate, D-glucosamine, pyruvate, L-proline, D-ribose, L-alanine, L-asparagine, and L-serine. The bacteria tested negative for indole production, acetoin production, lysine decarboxylase, ornithine decarboxylase, amylase, agarose, gelatinase and κ-carrageenase productions, and is incapable of assimilating D-fructose, sucrose, melibiose, lactose, N-acetylglucosamine, succinate, citrate, aconitate, meso-erythritol, γ-aminobutyrate, L-tyrosine, D-sorbitol, DL-malate, α-ketoglutarate, trehalose, gluconate, δ-aminovalate, cellobiose, L-glutamate, putrescine, propionate, amygdalin, arabinose, D-galacturonate, glycerate, D-raffinose, rhamnose, salicine, DL-lactate, L-arginine, L-citrulline, glycine, histidine, and L-ornithine. The G+C content of DNA is 42.1%. Estimated genome size is 3.4 Mb on the basis of genome sequencing.

## Supporting information

S1 Fig A broad NJ tree on the basis of 16S rRNA gene sequences. (PDF)

S2 Fig A broad Split network tree based on concatenated sequences of eight gene loci (*gapA*, *gyrB*, *ftsZ*, *mreB*, *pyrH*, *recA*, *rpoA*, and *topA*). (PDF)
