# Correction: *Vibrio aphrogenes* sp. nov., in the Rumoiensis clade isolated from a seaweed

**DOI:** 10.1371/journal.pone.0189555

**Published:** 2017-12-07

**Authors:** Mami Tanaka, Shoko Endo, Fumihito Kotake, Nurhidayu Al-saari, A. K. M. Rohul Amin, Gao Feng, Sayaka Mino, Hidetaka Doi, Yoshitoshi Ogura, Tetsuya Hayashi, Wataru Suda, Masahira Hattori, Isao Yumoto, Toko Sawabe, Tomoo Sawabe, Toshiyoshi Araki

The strain number is missing in the description of *V*. *aphrogenes* in the first and second paragraphs under the subheading “Description of *Vibrio aphrogenes* sp. nov.” in the Results and discussion section. Please see the corrected paragraph below.

## Description of *Vibrio aphrogenes* sp. nov.

*V*. *aphrogenes* sp. nov. (aph.ro'ge.nes. Gr. n. aphros, foam; Gr. suff. -genes, producing; N.L. adj. aphrogenes, foam-producing, referring to gas formation of the strain).

Gram-negative, facultative anaerobic, non-motile rods isolated from surface of seaweed collected in Mie Prefecture in Japan. Colonies on ZoBell 2216E agar medium were cream or transparent white, round, and smooth on the edge. No flagellum was observed. Sodium ion is essential for growth. Growth occurs at NaCl concentrations of 1.0 to 10.0% and at temperatures between 4 and 40°C. *V*. *aphrogenes* tested positive for production of alginase, lipase and DNase, oxidase, catalase, gas production from D-glucose, arginine dihydrolase, and is able to assimilate D-glucose, D-mannitol, D-mannose, D-galactose, maltose, D-gluconate, fumarate, glycerol, acetate, D-glucosamine, pyruvate, L-proline, D-ribose, L-alanine, L-asparagine, and L-serine. The bacteria tested negative for indole production, acetoin production, lysine decarboxylase, ornithine decarboxylase, amylase, agarose, gelatinase and κ-carrageenase productions, and is incapable of assimilating D-fructose, sucrose, melibiose, lactose, N-acetylglucosamine, succinate, citrate, aconitate, meso-erythritol, γ-aminobutyrate, L-tyrosine, Dsorbitol, DL-malate, α-ketoglutarate, trehalose, gluconate, δ-aminovalate, cellobiose, L-glutamate, putrescine, propionate, amygdalin, arabinose, D-galacturonate, glycerate, D-raffinose, rhamnose, salicine, DL-lactate, L-arginine, L-citrulline, glycine, histidine, and L-ornithine. The G+C content of DNA is 42.1%. Estimated genome size is 3.4 Mb on the basis of genome sequencing. The type strain is JCM 31643^T^ = DSM 103759^T^ = CA-1004^T^.
