# Supplementary figures and images for: Vibrio aphrogenes sp. nov., in the Rumoiensis clade isolated from a seaweed
Source: PLoS One. 2017 Jun 29;12(6):e0180053. doi: 10.1371/journal.pone.0180053 (PMC5491122; doi:10.1371/journal.pone.0180053)

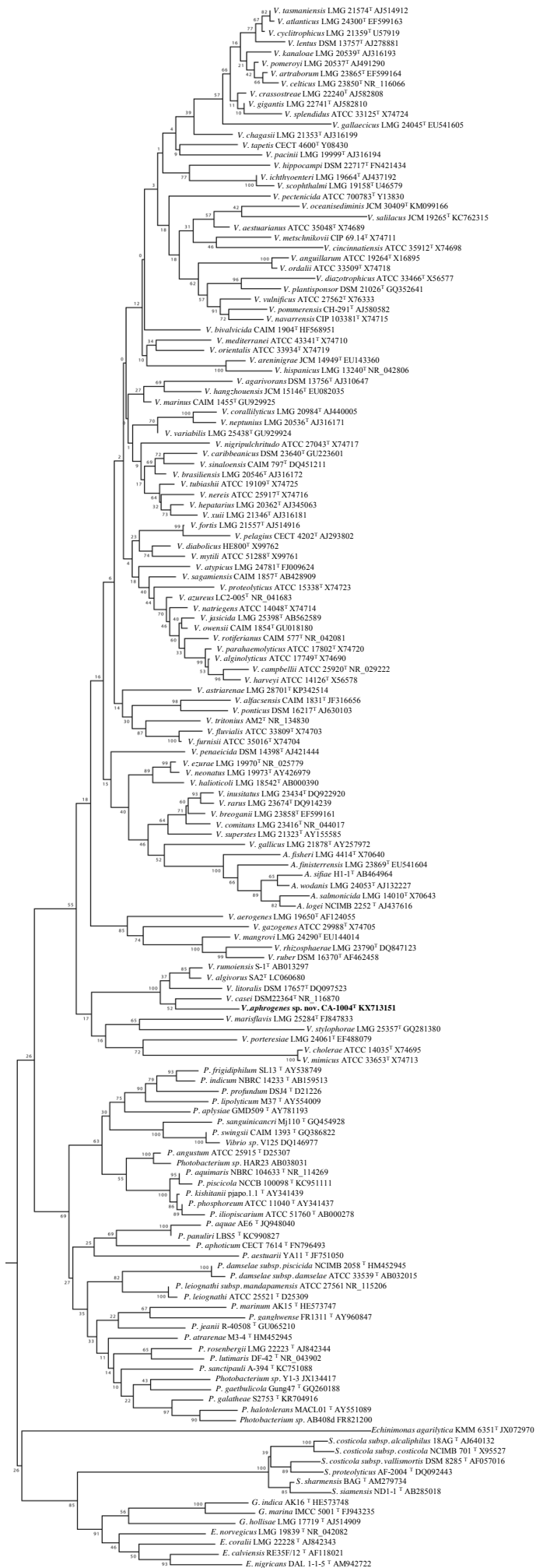

Supplement: S1 Fig — (PDF) [file pone.0180053.s001.pdf]

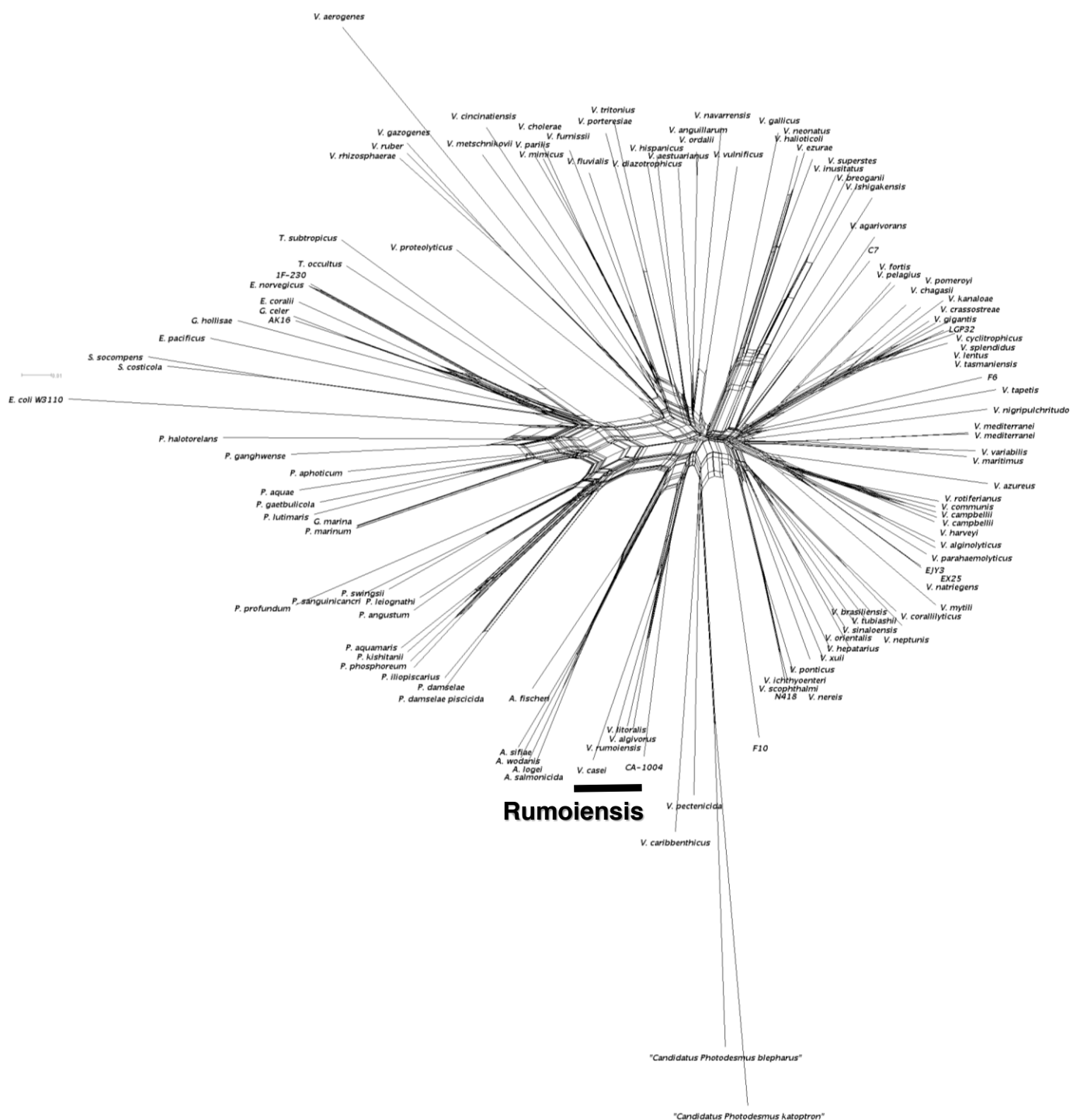

Supplement: S2 Fig — (PDF) [file pone.0180053.s002.pdf]
